# Gestational Weight Gain and Small for Gestational Age in Obese Women: A Systematic Review and Meta-Analysis

**DOI:** 10.1155/2023/3048171

**Published:** 2023-01-11

**Authors:** Wen Chen, Beiyi Li, Kexin Gan, Jing Liu, Yajing Yang, Xiuqin Lv, Huijuan Ma

**Affiliations:** ^1^Department of Anus and Intestine Surgery, Shijiazhuang People Hospital, Shijiazhuang 050000, Hebei, China; ^2^Department of Internal Medicine, Hebei Medical University, Shijiazhuang 050017, Hebei, China; ^3^Department of Endocrinology, Hebei General Hospital, Shijiazhuang 050017, Hebei, China; ^4^Graduate School of North China University of Science and Technology, Tangshan 063000, Hebei, China

## Abstract

**Objective:**

This systematic review and meta-analysis evaluates the relationship between gestational weight gain and the risk of small for gestational age in obese pregnant women.

**Methods:**

Studies were identified by searching the Web of Science, Embase, and PubMed databases up to June 30th, 2022. The meta-analysis was carried out to determine the risk of small for gestational age with gestational weight gain (GWG) below the 2009 Institute of Medicine (IOM) guidelines compared with within the guidelines in obese women. The Newcastle–Ottawa Scale was used to assess the methodological quality. The chi-squared test, *Q* test, and I^2^ test were used to evaluate statistical heterogeneity. Subgroup analyses were conducted, and publication bias was assessed by funnel plots and Egger's test. Sensitivity analyses were performed for three groups of obese people (I: BMI 30–34.9 kg/m^2^, II: BMI 35–39.9 kg/m^2^, and III: BMI ≥ 40 kg/m^2^) to examine the association of obesity and SGA.

**Results:**

A total of 788 references were screened, and 29 studies (*n* = 1242420 obese women) were included in the systematic review. Obese women who gained weight below the IOM guideline had a higher risk of SGA than those who gained weight within the guideline (OR = 1.27, 95% CI = 1.16–1.38, *Z* = 5.36). Both weight loss (<0 kg) and inadequate weight (0–4.9 kg) during pregnancy in obese women are associated with an increased risk of SGA (OR = 1.50, 95% CI = 1.37–1.64, *Z* = 8.82) (OR = 1.18, 95% CI = 1.14–1.23, *Z* = 8.06). The same conclusions were also confirmed for the three obesity classes (I: OR = 1.38, 95% CI = 1.29–1.47; II: OR = 1.39, 95% CI = 1.30–1.49; and III: OR = 1.26, 95% CI = 1.16–1.37). Subgroup analysis by country showed that GWG below guidelines in obese women of the USA and Europe was associated with risk for SGA (USA (OR = 1.30, 95% CI = 1.15–1.46), Europe (OR = 1.24, 95% CI = 1.11–1.40)) and not in Asia (OR = 1.17, 95% CI = 0.91–1.50).

**Conclusion:**

Our findings indicated that obese pregnant women who had weight loss or inadequate weight (0–4.9 kg) according to the IOM guideline had increased risks for SGA. Moreover, we also evaluated that gestational weight loss (<0 kg) in these pregnancies was associated with an increased risk for SGA compared with inadequate weight (0–4.9 kg) in these pregnancies. Therefore, the clinical focus should assist obese women to achieve GWG within the IOM guidelines to decrease the risk for SGA.

## 1. Introduction

Obesity has increased dramatically around the world in these past decades, and it is a public health problem. Obesity in pregnancy is often associated with adverse outcomes such as pregnancy-induced hypertension, preeclampsia, gestational diabetes mellitus (GDM), cesarean section, macrosomia, and neonatal asphyxia [1–3]. The Institute of Medicine (IOM) guideline revised the gestational weight gain in 2009 [4] and recommended that obese women should gain between 5 and 9 kg to obtain the best maternal and perinatal outcomes. However, the revision of the 2009 guideline did not provide recommended GWG for different classes of obesity.

Given the known relationship between gestational weight gain (GWG) above recommended and adverse perinatal outcomes, along with the long-term maternal health effects of obesity, physicians and women alike are exploring the possible benefits of weight loss during pregnancy-about 8% of all pregnant women reported attempting to lose weight, with the highest prevalence (13%) reported in obese women [5]. Moreover, the prevalence of actual weight loss increases with increasing obesity class, reaching as high as 15% in obesity class III [6, 7]. In this context, both prepregnancy BMI and GWG have been associated with maternal and fetal complications. However, there was no agreement on whether inadequate weight (0–4.9 kg) or weight loss (<0 kg) in obese women can contribute to improving neonatal outcomes or on the correct GWG to be reached to reduce these complications.

Some groups and meta-analyses suggested inadequate weight (0–4.9 kg) or weight loss (<0 kg) in obese women was associated with increase of SGA and low birth weight [8–12]. SGA and low birth weight not only increased the neonatal morbidity and mortality, but also some other chronic diseases such as type 2 diabetes, cardiovascular disease, and mental problems in adulthood [13–15]. However there is no agreement on inadequate weight (0–4.9 kg) or weight loss (<0 kg) in obese women. Some groups have suggested that appropriate management of inadequate weight (0–4.9 kg) or weight loss (<0 kg) can contribute to improving neonatal outcomes [6, 16, 17]. Therefore, the objective of this systematic review and meta-analysis was to assess the relationship between inadequate weight gain during pregnancy and the risk of SGA in obese women.

## 2. Materials and Methods

### 2.1. Data Source and Search Strategy

This review was registered in PROSPERO with the number CRD42022345753. We comprehensively searched the Web of Science, PubMed, and Embase to identify related articles published before June 30th, 2022, using keywords and MeSH headings for Pregnant Women, Pregnancy, Obesity, Gestational Weight Gain, Weight Gain, Infant, and Small for Gestational Age (Table S1). No language restriction was imposed. Reference lists were also assessed to acquire additional relevant articles. All relevant terms, including free-text terms and MeSH terms, were used in the literature search. All reference lists of the relevant reviews were hand-searched for additional relevant trials.

### 2.2. Eligibility Criteria

Studies were selected if they examined outcomes in women with BMI defined as obesity (BMI > 30 kg/m^2^, I: BMI 30–34.9 kg/m^2^, II: BMI 35–39.9 kg/m^2^, and III: BMI ≥ 40 kg/m^2^) assessed by self-reported or objective measurement before pregnancy, during pregnancy, or postpartum). Studies were included if the following criteria were met: (1) Population of singleton pregnancies. (2) The primary outcome assessed was the SGA (defined as birth weight less than the 10th percentile of birth weight for sex and gestational age). (3) Women with BMI defined as obesity (BMI > 30 kg/m^2^, I: BMI 30–34.9 kg/m^2^, II: BMI 35–39.9 kg/m^2^, and III: BMI ≥ 40 kg/m^2^) assessed by self-reported or objective measurement before pregnancy, during pregnancy, or postpartum) [18]. (4) Obese women who gained weight below the recommendation of the 2009 guideline including less than 0 kg (weight loss) and 0–4.9 kg (inadequate weight) compared the gaining with the guidelines (5–9 kg) [4].

Studies were excluded if they assessed a population that is not representative (diabetes women and women with second pregnancy), if the combined effects between obesity and weight gain in obese women were not examined, and if they were duplicate or secondary publications, opinion articles, reviews, guidelines, posters, conference papers, case reports, nonhuman studies, non-English articles, and without enough data.

### 2.3. Data Extraction and Quality Assessment

Two investigators (LJ and LBY) independently searched, selected, and extracted publications from the literature. Inconsistent data were discussed by the two investigators to reach consensus or evaluated by a third senior investigator (GKX). To assess the methodological quality of included studies, we used a modified version of the Newcastle–Ottawa Quality Scale. Two researchers (CW and LBY) independently evaluated the study quality and assigned the quality grades. Discrepancies were resolved by consensus of them and another researcher (GKX). The Newcastle–Ottawa Scale is composed of three categories: “Selection,” “Comparability,” and “Outcome.” Our modified Newcastle–Ottawa Scale excluded one item (“demonstration that outcome of interest was not present at the start of study”) of the “Selection” category since the lack of relevance for our meta-analysis. The elimination of the item left a maximum of three points for the “Selection” category. As our outcomes required follow-up until the end of pregnancy, another item, namely, “was follow-up long enough for an outcome to occur” under the “Outcome” category was excluded. A maximum of two points were awarded for this column. The two “most important confounding factors” of the “Comparability” criteria were selected on the basis of a prior knowledge of their association with GWG and each outcome. This modified Newcastle–Ottawa scale [19] ultimately conferred up to six points. Due to the shortage of validation studies that provided a cutoff score for rating low-quality studies, an arbitrary cutoff of four or fewer was used to categorize a study as “low quality.”

For the outcome (SGA), the points for confounding were allocated as follows: one point was allocated for controlling for parity, and an additional point for age, smoking, or diabetes mellitus (DM). We designated the lowest score for the outcome (SGA) without controlling all the items. The final comparability score was the minimum score that a study received for all the outcomes. “0” means no point awarded; “1” means one point awarded [9].

Two reviewers (CW and LBY) independently extracted the following data from full-text articles: name of the first author, year of publication, country of study, time span of the study (years), study setting, study design, characteristics of participants (including the population, source, and categories of BMI), confounding factors, and adjusted OR (95% CI). The results were verified again by another independent reviewer (CW) (Table 1).

### 2.4. Statistical Analysis

The multivariate-adjusted odds ratio (OR) and corresponding 95% CI reported in the studies were used to produce forest plots in our meta-analysis. Data were statistically analyzed using RevMan 5.3 software, and measured values were quantified using the weighted mean difference (WMD) with a 95% confidence interval (CI). Heterogeneity among different studies was quantified using I^2^. Heterogeneity was deemed statistically significant and insignificant when I^2^ values were >50% or ≤50%, respectively, and analyzed using random and fixed effects models, respectively. The reasons for heterogeneity were explored using subgroup analyses. Publication bias was quantified using funnel plots and Egger tests. Values with *P* < 0.05 were considered statistically significant, suggesting that publication bias was not excluded. Sensitivity analysis for outcome was performed for three groups of obese people (I: BMI 30–34.9 kg/m^2^, II: BMI 35–39.9 kg/m^2^, and III: BMI ≥ 40 kg/m^2^) to examine the effects of obesity and SGA.

## 3. Results

### 3.1. Literature Search

**Figure 1:**
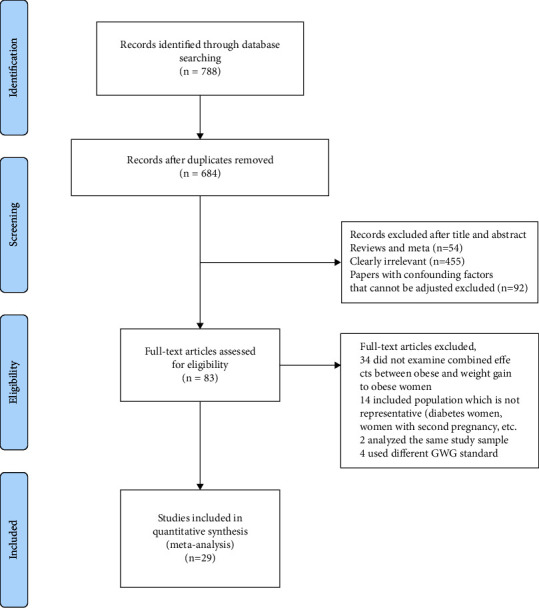
Flow of studies identified and included in the current meta-analysis.

The process of study identification and inclusion, and the reasons for exclusion are presented in Figure 1. A total of 788 studies were identified by the search. Following the removal of duplicates, 684 titles and abstracts were screened. Eighty-three studies were selected for full-text review, and 29 studies [8, 16, 17, 20–45], involving 1242420 obese pregnancies, met our eligibility criteria and were included in the systematic review. The kappa coefficient of agreement for included studies between the reviewers (LJ and LBY) was 0.99.

### 3.2. Study Characteristics

We got 29 articles [8, 16, 17, 20–45] including prospective [17, 29] and retrospective cohort studies [8, 16, 20–28, 30–45], built into our meta-analysis. The included studies reported on at least 1242420 obese pregnant women. At least 175247(14.11%) obese women were weight loss (<0 kg) and inadequate weight (0–4.9 kg) during the pregnancy compared with the recommendations of IOM guidelines. Twenty studies were American [8, 22–25, 27–31, 33, 34, 36, 37, 40–45], one was Japanese [32], one was from Lebanon [26], and seven were in Europe [16, 17, 20, 21, 35, 38, 39]. All but fifteen studies [8, 20, 21, 23, 24, 27, 28, 31, 33, 37–40, 45] investigated outcomes according to three groups of obese people. In addition, twenty-six studies [8, 16, 17, 20–22, 24–38, 40–42, 44, 45] also investigated outcomes for overall obesity except for three studies [23, 39, 43]. Eleven studies [8, 21, 22, 24, 28, 31–34, 40, 45] investigated outcomes for obese women who weight loss (<0 kg), and reported outcomes for obese women who inadequate weight (0–4.9 kg). Table 1 provides detailed information.

### 3.3. Quality Score

One study scored two points, three scored three points, six scored four points, twelve scored five points, and the others scored six points. The articles scoring below or equal to four points were regarded as “low quality” and would be subsequently involved in the sensitivity analysis (Table 2).

### 3.4. Outcomes

#### 3.4.1. Primary Outcomes

**Figure 2:**
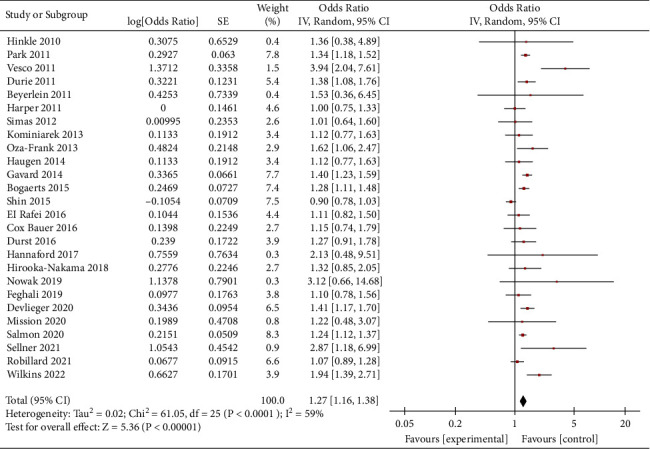
Forest plot of the association between weight gain below the guidelines in obese women and SGA.

**Figure 3:**
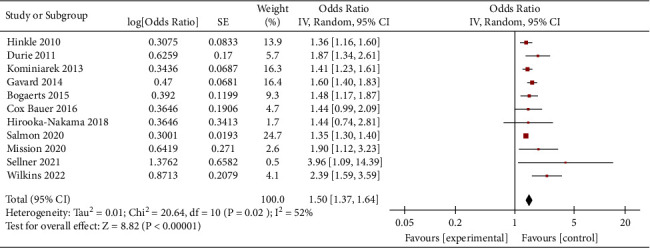
Forest plot of the association between weight loss in obese women and SGA.

**Figure 4:**
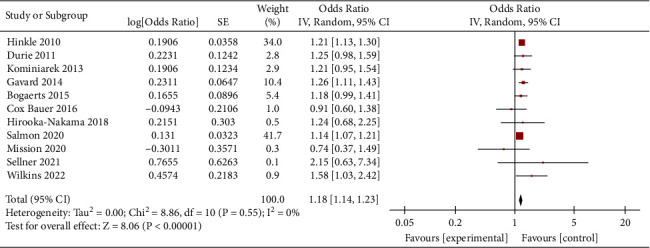
Forest plot of the association between inadequate weight in obese women and SGA.

Obese women who gained weight below the guideline recommendations had a higher risk of SGA than women who gained weight within the guidelines (OR = 1.27, 95% CI = 1.16–1.38, *Z* = 5.36, *P* < 0.00001; 26 studies). Heterogeneity as defined by the I^2^ statistics was high (I^2^ = 59%, *P* < 0.0001); therefore, a random effect model was used for the analysis (Figure 2). In obese women, weight loss (<0 kg) and inadequate weight (0–4.9 kg) during pregnancy were associated with an increased risk of SGA. (OR = 1.50, 95% CI = 1.37–1.64, *Z* = 8.82, *P* < 0.00001; 11 studies) (OR = 1.18, 95% CI = 1.14–1.23, *Z* = 8.06, *P* < 0.00001; 11 studies), as shown in Figures 3 and 4.

**Figure 5:**
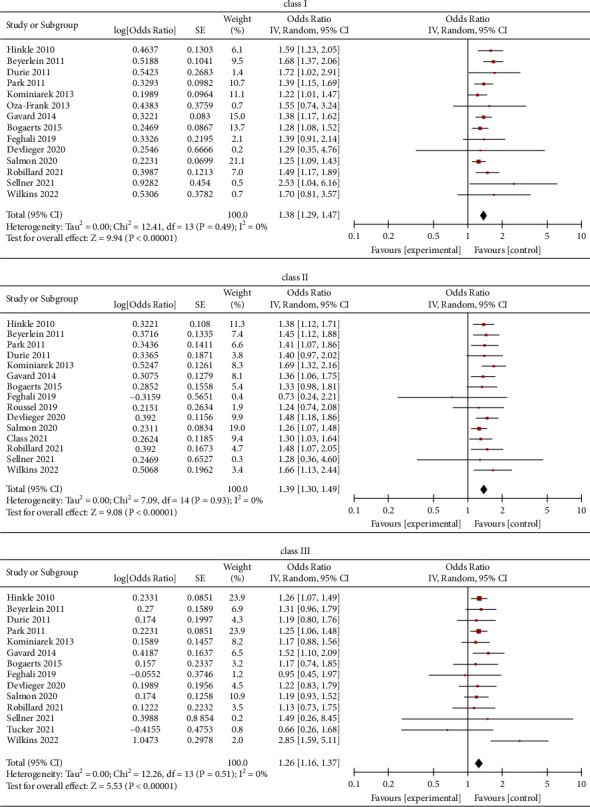
Forest plot of the association between weight gain below the guidelines in obese women of each class group and SGA.

Data from each class group showed the differences between the obese women who gained weight below the guidelines and those who gained weight within. Class I (OR = 1.38, 95% CI = 1.29–1.47, *Z* = 9.94, *P* < 0.00001; 14 studies); Class II (OR = 1.39, 95% CI = 1.30–1.49, *Z* = 9.08, *P* < 0.00001; 15 studies) Class III (OR = 1.26, 95% CI = 1.16–1.37, *Z* = 5.53, *P* < 0.00001; 14 studies) (Figure 5)

**Figure 6:**
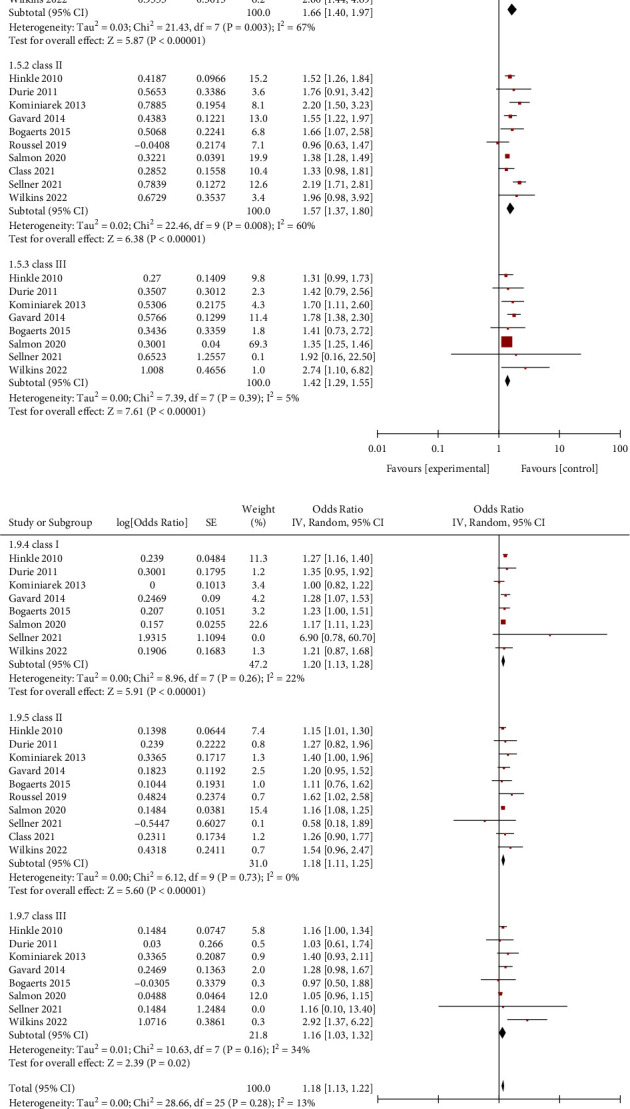
Forest plot of weight loss and inadequate weight with SGA for obese women of each class group. (a) Weight loss. (b) Weight inadequately.

**Figure 7:**
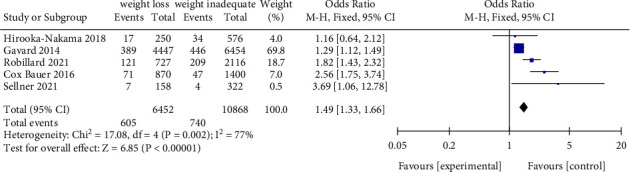
Forest plot of weight loss and inadequate weight with SGA for obese women.

**Figure 8:**
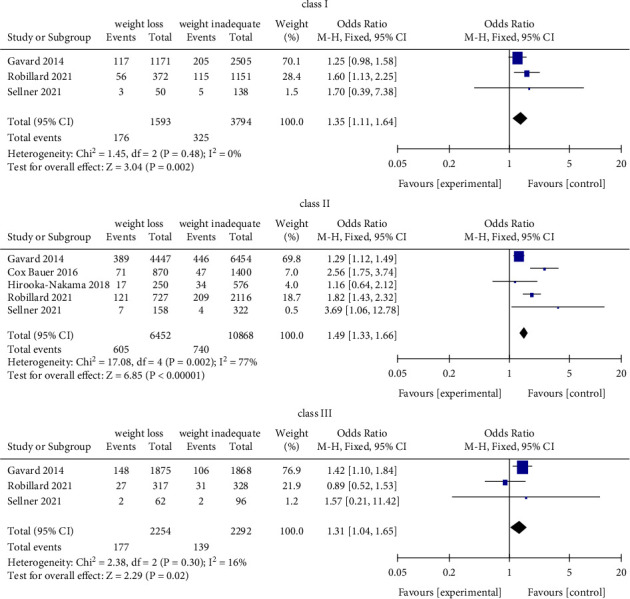
Forest plot of weight loss and inadequate weight with SGA for obese women of each class group.

The same results were identified by Class I, Class II, and Class III of obese women who weight loss (<0 kg) and inadequate weight (0–4.9 kg) during pregnancy (Figure 6). In addition, we found that gestational weight loss (<0 kg) was associated with an increased risk for SGA (OR = 1.49, 95% CI = 1.33–1.66, *Z* = 6.85, *P* < 0.00001; 5 studies), as compared with inadequate weight (0–4.9 kg) (Figures 7 and 8).

#### 3.4.2. Subgroup Analysis

**Figure 9:**
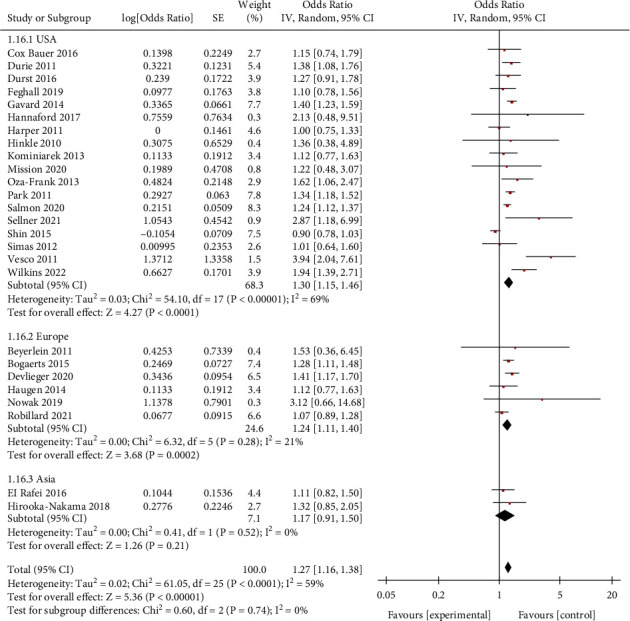
Forest plot of the association between weight gain below the guidelines in obese women of each country and SGA.

The results of subgroup analysis by countries showed that GWG below guidelines in obese women were associated with risk for SGA: USA (OR = 1.30, 95% CI = 1.15–1.46, *Z* = 4.27, *P* < 0.0001; 18 studies) and Europe (OR = 1.24, 95% CI = 1.11–1.40, *Z* = 3.68, *P*=0.0002; 6 studies), with no statistically significant result for Asia. (OR = 1.17; 95% CI = 0.91–1.50; *Z* = 1.26, *P*=0.21; 2 studies) (Figure 9).

#### 3.4.3. Sensitivity Analyses and Publication Bias

Sensitivity analysis was used to evaluate the stability of the results. The sensitivity analysis indicated, compared with the original pooled OR, excluding the ten studies assessed as “low quality” also resulted in a similar (OR = 1.31; 95% CI = 1.25–1.38; *Z* = 10.37, *P* < 0.00001).

**Figure 10:**
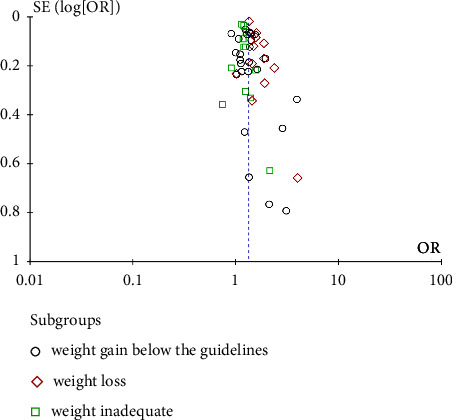
Funnel plots for effect of weight gain below the guidelines with SGA for overall obesity, inadequate weight, and weight loss.

No more evidence of publication bias showed in the funnel plots for the overall obesity, weight loss, and inadequate weight group (Figure 10).

## 4. Discussion

Our meta-analysis demonstrates that obese women who gained weight below the guideline recommendations had more risks of SGA than those of gained weight within the guidelines. These data covered not only the population of overall obese women but all three classes of obesity of pregnant women. These results were similar to prior systematic reviews [9–11, 46]. However, these studies did not account for the differing socioeconomic, lifestyle, and racial backgrounds of patients. The repercussions of weight loss in obese gravida may varied based on race and socioeconomic classes, so studying these topics in diverse patient populations was important. Moreover, in our study, we also evaluated gestational weight loss in obese pregnant women was associated with an increases risk for SGA, compared with inadequate weight (0–4.9 kg). Of the 29 articles we selected, only five provided detailed information on the number of pregnancies at risk of weight loss (<0 kg) and inadequate weight (0–4.9 kg) for SGA. Therefore, our study added a subgroup analysis of race and found that obese women who gain weight below the guideline in the United States and Europe were associated with a higher risk for SGA, but not in Asia because the USA and Europe had the greatest prevalence of overweight and obesity [47, 48]. Asia women were more likely to be underweight than those in the USA and Europe [32].

Obesity during pregnancy is associated with a myriad of adverse outcomes such as preeclampsia, labour induction, postpartum haemorrhage, cesarean delivery, and preterm birth [49, 50]. Therefore, more obese women in the USA and Europe attempted to lose weight during pregnancy [51]. Our study also analyzed that not only weight loss (<0 kg) but also inadequate weight (0–4.9 kg) in obese pregnant women were associated with an increased risk of SGA. Our findings in these meta-analyses were also in line with the findings of a previous meta-analysis [10, 11]. Moreover, our results were identified by Class I, Class II, and Class III of obese women. The mechanism of weight gain within the guideline range during pregnancy contributes to SGA may be that the lack of maternal nutrition can lead to the placental vascular development change and barrier thickness increases, thus resulting in reduced glucose, amino acid, and lipid transport, as well as chronic hypoxia, which ultimately affected the fetus normal growth and development process.

### 4.1. Strength and Limitations

The strength of this systematic review included the comprehensiveness of the search strategies in three databases. We performed a careful quality assessment using a modified Newcastle–Ottawa scale. Sensitivity analyses corroborated the robustness of our findings and argued in favour of their validity. Importantly, we addressed the evidence for each obesity class. All included studies were adjusted for multiple important confounders, and all but ten studies were of high quality.

However, this meta-analysis has several limitations. First, it lacked studies from developing countries. The studies that met our inclusion criteria originated predominantly from United States. Hence, more research is needed from diverse populations to be able to generalize our findings. Second, some variables may also have influenced findings, such as maternal ethnicity, behavioral factors (diet, physical activity, and smoking), socioeconomic status, and women with prenatal complications, although some studies did adjust for these variables or excluded women with preexisting complications from their analysis. Third, the limits are related to the precision of self-reported GWG, the no possibility to obtain data on the dietary advice and dietary compliance of the women and on the long-term outcomes of neonates.

## 5. Conclusions

Our findings indicated that obese pregnant women who weight loss (<0 kg) and inadequate weight (0–4.9 kg) below the IOM guideline had increased risks for SGA. Therefore, the clinical focus should intensify efforts to assist obese women to achieve GWG within the IOM guidelines to decrease the risk for SGA. We also found that gestational weight loss in these pregnancies was associated with an increased risk for SGA compared with weight inadequate. These findings underline the importance considering the IOM guidelines in terms of gestational weight gain taking into consideration the different classifications of obese women.

## Figures and Tables

**Table 1:** Characteristics of cohort studies included in meta-analyses of SGA in obese pregnant women who gained inadequate weight compared with those gained within the IOM.

Study	Race	Period	Study design	Population	Setting	Sample size (overall)	Sample size in each obesity class	Source of BMI	Adjustment by factors	Adjusted OR (95% CI)
Beyerlein et al. (2011) [20]	Bavarian (Europe)	2000–2007	Retrospective cohort study	Women with singleton deliveries in Bavarian obstetric units	Bavarian Working Group on Clinical Quality Assessment	Total *n* of samples = 709575; 2214 (only those weight loss); total *n* of obese women = 73128	Obese Class I: 49240	Measured	Gestational and pregestational diabetes; smoking during pregnancy; gender parity; maternal age	OR 1.36 (1.20–1.55)
										Obese Class I
							Obese Class II: 16673			1.68 (1.37–2.06)
										Obese Class II
							Obese Class III: 7215			1.45 (1.12–1.89)
										Obese Class III 1.31 (0.96–1.79)

Bogaerts et al. (2015) [21]	Belgium	2009–2011	Retrospective cohort study	All liveborn singleton term (37 weeks of gestation or greater) births in obese women in Flanders (the northern part of Belgium)	The Flemish Study Center for Perinatal Epidemiology database	Total *n* of samples = 18065; 8467 (5120 gained within; 3347 gained below guidelines); total *n* of obese women = 18053	NR	Self-reported	Maternal and gestational age; parity	OR 1.34 (1.18–1.51)
										Obese Class I
										1.28 (1.08–1.52)
										Obese Class II
										1.33 (0.98–1.79)
										Obese Class III
										1.17 (0.74–1.87)

Cox Bauer et al. (2016) [22]	United States	1/2008–12/2013	Retrospective cohort study	Women in the study who gave birth at any of 12 hospitals in a single, regional healthcare system	Women in the study who gave birth at any of 12 hospitals in a single, regional health-care system	Total *n* of samples = 17244; 4703 (3833 gained within; 870 gained below guidelines); total *n* of obese women = 10734	NR	Calculated	NR	OR 1.53 (1.32–1.76)

Class et al. (2021) [23]	United States	2000–2015	Retrospective cohort study	The sample was limited to primiparous singleton pregnancies	Electronic medical records (EMR) at the University of Illinois Hospital, Chicago	Total *n* of samples = 25604; total *n* of obese women = 15753	Obese Class I: 7191	Self-reported	Maternal age, year of birth, gestational age at delivery, GD, and PIH	Obese Class II 1.3 (1.04–1.63)
							Obese Class II: 5512			
							Obese Class III: 4102			

Devlieg-er et al. (2020) [16]	Belgium	2009–2014	Retrospective cohort study	Women delivering a singleton term (≥37 weeks) live birth	The data from Flemish study Center for Perinatal Epidemiology (SPE)	Total *n* of samples = 337590; 17345 (10381 gained within; 6964 gained below guidelines); total *n* of obese women = 36791	Obese Class I: 26488	Measured	Parity, maternal age, mode of conception (spontaneous and assisted), gestational age, and year of delivery	OR 1 (0.75–1.33)
										Obese Class I
										1.29 (1.12–1.47)
							Obese Class II: 7748			Obese Class II
										1.48 (1.18–1.86)
							Obese Class III: 2555			Obese Class III
										1.22 (0.83–1.79)

Durie et al. (2011) [24]	New York	2004–2008	Retrospective cohort study	Singleton live birth ≥20 weeks	Five Lakes Region Perinatal Data System	Total *n* of samples = 73977; 7575 (2850 gained within; 4675 gained below); total *n* of obese women = 17517	Obese Class I: 9389	Prepregnancy BMI was calculated using the patient-reported prepregnancy weight and height as documented on the birth certificate	Maternal race/ethnicity; maternal education; tobacco use; nulliparity; chronic hypertension; preexisting diabetes	OR 1.38 (1.08–1.75)
										Obese Class I
										1.72 (1.02–2.92)
							Obese Class II: 4728			Obese Class II
										1.40 (0.97–2.02)
							Obese Class III: 3400			Obese Class III
										1.19 (0.80–1.75)

Durst et al. (2016) [25]	United States	2000–12/2014	Retrospective cohort study	Obese women with singleton pregnancies delivering	University of Alabama at Birmingham	Total *n* of samples = 5651; 2830 (1352 gained within; 1478 gained below guidelines); total *n* of obese women = 5651	Obese Class III: 1558	Maternal BMI was determined from the height and weight recorded at the first prenatal visit	Adjusted for prior cesarean, age, race, parity, gestational age, payor status, and tobacco use	OR 3.94 (2.04–7.61)

El Rafei et al. (2016) [26]	Lebano-n	2001–2012	Retrospective cohort study	Singleton livebirths with gestational age between 28 and 42 weeks	The NCPNN network	Total *n* of samples = 170428; 4122 (2310 gained within; 1812 gained below guidelines); total *n* of obese women = 11274	NR	Calculated	Body mass index (kg/m^2^), maternal education, maternal age, paternal age, smoking during pregnancy, newborn sex, consanguinity, and parity	OR 1.01 (0.64–1.61)

Feghali et al. (2019) [27]	United States	2012–2014	Retrospective cohort study	We included women who had at least one measured weight between 24 and 28 weeks of gestation and documented prenatal care in the general obstetrics, midwifery, and maternal–fetal medicine clinics in our hospital system	Magee-Womens Hospital of UPMC (University of Pittsburgh Medical Center, Pittsburgh, Pennsylvania)	Total *n* of samples = 5814; total *n* of obese women = 2875	Obese Class I: 1551	Measured	NR	OR 1.62 (1.06–2.46)
										Obese Class I
										1.40 (0.90–2.14)
							Obese Class II: 748			Obese Class II
										0.73 (0.24–2.21)
							Obese Class III: 576			Obese Class III
										0.95 (0.45–1.97)

Gavard et al. (2014) [28]	United States	2002–2008	Population-based historical cohort study	66 010 obese pregnant women in Missouri delivering liveborn, singleton	Data from the Missouri maternally linked birth and fetal death certificate registry	Total *n* of samples = 66010; 25976 (15075 gained within; 10901 gained below guidelines); total *n* of obese women = 66010	Obese Class I: 36568	Self-reported	Maternal age, race, socioeconomic status, smoking, parity, cardiac disease, renal disease, chronic hypertension, and preeclampsia	OR 1.12 (0.77–1.63)
										Obese Class I
										1.38 (1.17–1.62)
							Obese Class II: 17195			Obese Class II
										1.36 (1.06–1.75)
							Obese Class III: 12247			Obese Class III
										1.52 (1.10–2.09)

Hannaford et al. (2017) [29]	United States	12/2008–4/2012	Prospective cohort study	Women with singleton gestations	Obstetrics and Gynecology, Washington University	Total *n* of samples = 1120; 90 (49 gained within; 41 gained below guidelines); total *n* of obese women = 258	NR	Prepregnancy maternal BMI was calculated based on patient reported height and weight	smoking, chronichypertension, and macrosomia	OR 1.40 (1.23–1.58)

Harper et al. (2011) [30]	Missou-ri	1989–2005	Retrospective cohort study	Women who were primiparous, who had singleton gestations, who were 520 years old, and who delivered at 24–44 weeks' gestation	Swedish Medical Birth Registry	Total *n* of samples = 76682; 1778 (1090 gained within; 688 gained below guidelines); total *n* of obese women = 6279	NR	Self-reported	Maternal age; race; smoking/alcohol use during pregnancy; medicaid use; prepregnancy BMI; chronic hypertension; DM; renal disease	OR 1.28 (1.11–1.47)

Haugen et al. (2014) [17]	Norway	1998–2008	Prospective cohort study	Women who delivered a singleton liveborn child during gestational weeks 37–42 and recruited from all over Norway	Norwegian Mother and Child Cohort Study (MoBa), conducted from the Norwegian Institute of Public Health	Total *n* of samples = 56101; 1931 (1054 gained within; 877 gained below guidelines); total *n* of obese women = 4963	Obese Class I: 3680	Self-reported	Maternal age; maternal height; maternal education; gestational weight; smoking, diabetes; separate analysis for nulliparous and parous	OR 0.9 (0.78–1.03)
							Obese Class II: 976			
							Obese Class III: 307			

Hinkle et al. (2010) [31]	Six unspecified states, United State	2004–2006	Retrospective cohort study	Obese nonhispanic white, nonhispanic black, and hispanic women with available data from a prenatal and postpartum visit, singleton term births	Primarily the Special Supplemental Nutrition Program for Women, Infants, and Children (WIC)	Total *n* of samples = 122327; 51328 (26437 gained within; 24891 gained below guidelines) total *n* of obese women = 122327	Obesity Class I: 64717	Self-reported	Education; gestational age; infant sex; marital status; maternal height; race/ethnicity; smoking	OR 1.11 (0.82–1.50)
										Obese Class I
										1.59 (1.23–2.05)
							Obesity Class II: 33156			Obese Class II
										1.38 (1.12–1.71)
							Obesity Class III: 24454			Obese Class III
										1.25 (1.06–1.48)

Hirooka-Nakama et al. (2018) [32]	Japanese	1/2013–12/2013	Retrospective cohort study	Women who delivered singleton term live births between	Approximately 280 secondary and tertiary hospitals participated in the JSOG successive pregnancy birth registry system	Total *n* of samples = 64027; 1387 (560 gained within; 827 gained below guidelines); total *n* of obese women = 1840	NR	Calculated from the self-reported prepregnancy weight and height	Parity, maternal age, smoking, and gestational age, and generalized estimating equations (GEE)	OR 1.15 (0.74–1.81)

Komini-arek et al. (2013) [33]	United States	2002–2008	Retrospective cohort study	20950 obese women with a singleton, term live birth from the consortium on safe labor	12 institutions (19 hospitals) across nine ACOG districts in the United States	Total *n* of samples = 20950; 7823 (3613 gained within; 4210 gained below guidelines); total *n* of obese women = 20950	Obese Class I: 11984	Self-reported	Gestational age; insurance; marital status; maternal age; parity; race/ethnicity; smoking	
										OR 1.41 (1.23–1.61)
										Obese Class I
										1.22 (1.01–1.47)
							Obese Class II: 5307			Obese Class II
										1.69 (1.32–2.16)
							Obese Class III: 3659			Obese Class III
										1.53 (1.15–2.03)

Mission et al. (2020) [34]	United States	10/2012–8/2014	Retrospective cohort study	All women with obesity (defined as a prepregnancy BMI >30 kg/m^2^), singleton gesta tions, onset of prenatal care before 24 weeks, and no history of pre-GDM at UPMC Magee-Womens Hospital (University of Pittsburgh, Pittsburgh, PA)	Magee Obstetric Medical and Infant (MOMI) database, which includes variables for all births at UPMC Magee-Womens Hospital in Pittsburgh, PA	Total *n* of samples = 2698; 1211 (539 gained within; 672 gained below guidelines); total *n* of obese women = 2698	Obese Class I: 1446	Measured	NR	OR 1.22 (0.49–3.07)
							Obese Class II: 701			
							Obese Class III: 551			

Nowak et al. (2019) [35]	Poland	11/2006–11/2007	Retrospective cohort study	(1) Singleton pregnancy	In the Obstetrics and Perinatology Department at Jagiellonian University Hospital in Cracow, Poland	Total *n* of samples = 474; total *n* of obese women = 27	NR	Calculated using maternal weight before pregnancy and height	NR	OR 2.13 (0.48–9.51)
				(2) No maternal chronic diseases,						
				(3) No congenital fetal abnormalities						

Oza-Frank et al. (2013) [36]	United States	1959–1965	Prospective cohort study	Obese women at their first prenatal visit, singleton	12 urban U.S. sites	Total *n* of samples = 11203; 1899 (632 gained within; 1267 gained below guidelines); total *n* of obese women = 2789	Obese Class I: 1968	Self-reported	Maternal age, parity, pre-pregnancy BMI, race/ethnicity, socioeconomic status, smoking	OR 1.32 (0.85–2.06)
							Obese Class II: 588			Obese Class I
							Obese Class III: 233			1.55 (0.74–3.23)

Park et al. (2011) [37]	Florida, United States	2004–2007	Retrospective cohort study	Women aged 18–40 years with a singleton full term (37–41 week s), live birth; available information for prepregnancy BMI, gestational weight change, and LGA, or SGA status	Florida live birth certificate	Total *n* of samples = 570672; 37090 (17350 gained within; 19740 gained below); total *n* of obese women = 101590	NR	Prepregnancy BMI data from Florida birth certificates	Maternal age; race/ethnicity; education; marital status; smoking status during pregnancy; parity; WIC program participation; gestational age	OR 3.1 (0.66–14.68)
										Obese Class I
										1.39 (1.15–1.69)
										Obese Class II
										1.41 (1.07–1.86)
										Obese Class III
										1.25 (1.03–1.51)

Robillard et al. (2021) [38]	Europe	1/2001–12/2019	Retrospective cohort study	All consecutive term (37–42 weeks gestation) singleton pregnancies (>21 weeks) live birth pregnancies delivered in the maternity	The hospital records of all women delivered at the maternity of the University South Reunion Island	Total *n* of samples = 61764; 3464 (1941 gained within; 1151 gained below; 372 weight loss); total *n* of obese women = 11097	Obese Class I: 6644	Measured	NR	OR 1.10 (0.78–1.56)
										Obese Class I
										1.49 (1.17–1.89)
							Obese Class II: 2938			Obese Class II
										1.48 (1.06–2.05)
							Obese Class III: 1515			Obese Class III
										1.13 (0.72–1.75)

Roussel et al. (2019) [39]	France	1/2006–12/2015	Retrospective cohort study	Singleton pregnancy, term delivery (between 37 and 41 weeks of gestation), and a BMI comprised between 35 and 40 kg/m^2^	Two hospitals providing level III (Rouen University Hospital) and II (Belvedere General Hospital)	Total *n* of samples = 1537; 996 (424 gained within; 370 gained below guidelines; 202 weight loss); total *n* of obese women = 1537	Obese Class II: 1537	Maternal height and weight before pregnancy and weight at delivery from the flemish study center for perinatal epidemiology (SPE)	Maternal age, parity, ethnicity, chronic hypertension, preexisting diabetes mellitus, and tobacco use	Obese Class II 1.24 (0.74–2.06)

Salmon et al. (2020) [40]	United States	2014	Retrospective cohort study	Women were included in the present study if they delivered a singleton liveborn infant at term (37–40 weeks) gestation, had a prepregnancy BMI ≥30 kg/m^2^, and had no missing data on maternal height, prepregnancy weight, and weight at deliver	Birth certificate data from the 2014 United States (US) Natality Files	Total *n* of samples = 3998076; 289206 (150189 gained within; 86928 gained below; 52089 weight loss); total *n* of obese women = 642096	Obese Class I: 355923	Measured	Maternal age, maternal education, marital status, parity, medical insurance, maternal race, prepregnancy diabetes, and gestational diabetes, and smoking	OR 1.41 (1.16–1.7)
										Obese Class I
										1.25 (1.09–1.43)
							Obese Class II: 169000			Obese Class II
										1.26 (1.07–1.5)
							Obese Class III: 117173			Obese Class III
										1.19 (0.93–1.52)

Sellner et al. (2021) [8]	United States	9/2013–8/2017	Retrospective cohort study	1428 women with prepregnancy BMI ≥30 kg/m^2^ who received prenatal care at a large Medicaid clinic	Electronic medical record in Department of Obstetrics and Gynecology, Baylor college of Medicine	Total *n* of samples = 890; 416 (176 gained within; 161 gained below; 79 weight loss); total *n* of obese women = 890	Obese Class I: 484	Measured	Age, race/ethnicity, nutritional education, participation in group prenatal care, and medical conditions	OR 1.35 (0.94–1.93)
										Obese Class I
										2.53 (1.04–6.16)
							Obese cClass II: 218			Obese Class II
										1.28 (0.36–4.6)
							Obese Class III: 188			Obese Class III
										1.49 (0.26–8.45)
Shin et al. (2015) [41]	United States	2004–2011	Retrospective cohort study	Women from the PRAMS (an ongoing surveillance project of the Centers for Disease Control and Prevention (CDC) and state health departments of 40 U.S. states and New York City)	Pregnancy Risk Assessment Monitoring System (PRAMS)	Total *n* of samples = 219868; total *n* of obese women = 42963	NR	NR	Maternal age; gestational age; smoking; maternal education; family income; marital status	OR 1.24 (1.13–1.37)

Simas et al. (2012) [42]	United States	4/2006–3/2010	Retrospective cohort study	Women who delivered singleton, live birth, and nonanomalous neonates	University of Massachusetts (UMass) Memorial Healthcare automated electronic labour and delivery data	Total *n* of samples = 11203; 882 (424 gained within; 458 gained below guidelines); total *n* of obese women = 2313	NR	(1) Self-reported prepregnancy weight as recorded in the woman's pre natal record, (2) weight self-reported by the women upon admission for delivery, (3) measured weight at first prenatal visit as recorded in her prenatal record	Diabetes; marital status; hypertension; parity; race/ethnicity and smoking	OR 1.07 (0.9–1.28)

Tucker et al. (2021) [43]	Florida, USA	7/2013–12/2017	Retrospective cohort study	Patients who delivered a singleton at term (>37 weeks and 0 days) with a BMI >40 kg/m^2^	Electronic medical record in Duke University Hospital or Duke Regional Hospital	Total *n* of samples = 374; 230 (101 gained within; 129 gained below); total *n* of obese women = 374	Obese Class III: 374	Measured	After controlling for entry BMI and gestational age at delivery	Obese Class III
										0.66 (0.26–1.66)

Vesco et al. (2011) [44]	Oregon and Washington, United States	1/2000–12/2005	Retrospective cohort study	Women with singleton live births ≥37 weeks, who delivered with in Kaiser Permanente Northwest with measured maternal weight between 6 months before pregnancy and 12 weeks; measured weight within the 2 weeks before delivery; documented height	Electronic medical records in Kaiser Permanente Northwest	Total *n* of samples = 12076 887 (513 gained within; 374 gained below guidelines); total *n* of obese women = 2080	NR	Measured	Parity; maternal age; gestational age at delivery; tobacco use during the last trimester of pregnancy; Medicaid enrollment	OR 2.87 (1.18–6.99)

Wilkins et al. (2022) [45]	United States	2009–2012	Retrospective cohort study	Singleton pregnancies >35 weeks with prepregnancy obesity	Kaiser Permanente Northern California	Total *n* of samples = 17563; total *n* of obese women = 17563	Obese Class I: 2459	NR	Age, race, parity, gestational weeks, start of prenatal care, pregestational diabetes, GDM, neonate, education, substance use, prepregnancy BMl, rior chronic hypertension, and hypertension in pregnancy	OR 1.94 (1.39–2.71)
										Obese Class I
										1.7 (0.81–3.59)
							Obese Class II: 3688			Obese Class II
										1.`66 (1.13–2.46)
							Obese Class III: 4742			Obese Class III
										2.85 (1.59–5.09)

NR, not report.

**Table 2:** Quality assessment of included studies using the modified Newcastle–Ottawa scale in systematic review of gestational weight gain below compared with within the 2009 Institute of Medicine Guideline in obese women and SGA.

Study	Modified Newcastle–Ottawa scale					
	Selection			Comparability of cohorts (maximum: 2)	Outcome assessment of outcomes (maximum: 1)	Overall scores
	Representativeness of exposed cohorts (maximum: 1)	Selection of nonexposed cohorts (maximum: 1)	Ascertainment of exposures (maximum: 1)			
Wilkins et al. (2022) [45]	1	1	1	2	1	6
Tucker et al. (2021) [43]	1	1	1	1	1	5
Sellner et al. (2021) [8]	0	1	0	1	1	3
Robillard et al. (2021) [38]	1	1	1	0	0	3
Class et al. (2021) [23]	0	1	0	1	1	4
Salmon et al. (2020) [40]	1	1	1	2	1	6
Mission et al. (2020) [34]	1	1	1	2	1	6
Devlieger et al. (2020) [16]	1	1	1	1	1	5
Roussel et al. (2019) [39]	0	1	0	2	1	4
Feghali et al. (2019) [27]	1	1	1	1	1	4
Nowak et al. (2019) [35]	0	1	1	1	1	4
Hirooka-Nakama et al. (2018) [32]	0	1	0	2	1	4
Hannaford et al. (2017) [29]	1	1	1	1	1	5
Durst et al. (2016) [25]	1	1	1	1	1	5
Cox Bauer et al. (2016) [22]	1	1	1	1	1	5
El Rafei et al. (2016) [26]	1	1	0	2	1	5
Shin et al. (2015) [41]	1	1	0	2	1	5
Bogaerts et al. (2015) [21]	1	1	0	2	1	5
Gavard et al. (2014) [28]	1	1	0	2	1	5
Haugen et al. (2014) [17]	1	1	0	2	1	5
Oza-Frank et al. (2013) [36]	1	1	0	2	1	5
Simas et al. (2012) [42]	0	1	0	2	1	4
Vesco et al. (2011) [44]	0	1	1	2	1	5
Park et al. (2011) [37]	1	1	1	2	1	6
Durie et al. (2011) [24]	1	1	1	2	1	6
Harper et al. (2011) [30]	1	0	1	1	0	3
Beyerlein et al. (2011) [20]	1	1	1	2	1	6
Hinkle et al. (2010) [31]	1	0	1	0	0	2

## Data Availability

The data described in this article can be freely and openly accessed from the original published articles in the database.

## Supplementary Materials

Supplementary Materials Table S1: summary of the medical search strategy for this article.
