# Supplementary material for: Gestational Weight Gain and Small for Gestational Age in Obese Women: A Systematic Review and Meta-Analysis
Source: Int J Endocrinol. 2023 Jan 11;2023:3048171. doi: 10.1155/2023/3048171 (PMC9848811; doi:10.1155/2023/3048171)
Supplement: Supplementary Materials — Table S1: summary of the medical search strategy for this article. [file 3048171.f1.pdf]

This is a summary of the overall search strategy of the article, by searching for the relevant keywords and subject words and multiple combinations, to search out the literature we want.

Supplementary Table 1: Medline Search Strategy

1. Pregnant Women[MeSH Terms]
2. Pregnant Woman [tiab]
3. Woman, Pregnant[tiab]
4. Women, Pregnant[tiab]
5. 1 or 2 or 3 or 4
6. Pregnancy [MeSH Terms]
7. Pregnancies [tiab]
8. Gestation [tiab]
9. 6 or 7 or 8
10. 5 or 9
11. Obesity [MeSH Terms]
12. Obese [tiab]
13. 11 or 12
14. 10 and 13
15. Gestational Weight Gain [MeSH Terms]
16. Weight Gain, Gestational [tiab]
17. Pregnancy Weight Gain [tiab]
18.Weight Gain, Pregnancy[tiab]
19. Maternal Weight Gain[tiab]
20. Weight Gain, Maternal[tiab]
21.Postpartum Weight Retention[tiab]
22.Weight Retention[tiab]
23.Postpartum[tiab]
24. 15 or 16 or 17 or 18 or 19 or 20 or 21 or 22 or 23
25.Weight Gain[MeSH Terms]
26.Gain, Weight [tiab]
27.Gains, Weight [tiab]

28.Weight Gains [tiab]

29.24 or 25 or 26 or 27

30.24 or 29

31.14 and 30

32.Infant, Small for Gestational Age[MeSH Terms]

33.SGA [tiab]

34.32 or 33

35.31 and 34
